# Supplementary material for: Analysis of PPARγ Signaling Activity in Psoriasis
Source: Int J Mol Sci. 2021 Aug 10;22(16):8603. doi: 10.3390/ijms22168603 (PMC8395241; doi:10.3390/ijms22168603)
Supplement: Supplementary file 1 [file ijms-22-08603-s001.zip › Supplemental materials_Analysis of PPARg signaling activity in psoriasis/Pathway models/Models images and html files/Model of PPARG related pathways in psoriasis (short version)/6804.html]

KRAS


# Protein KRAS

|  |  |
| --- | --- |
| URN | urn:agi-llid:3845 |
| Total Entities | 0 |
| Connectivity | 4266 |
| Name | KRAS |
| Description | Kirsten rat sarcoma viral oncogene homolog |
| Notes | This gene, a Kirsten ras oncogene homolog from the mammalian ras gene family, encodes a protein that is a member of the small GTPase superfamily. A single amino acid substitution is responsible for an activating mutation. The transforming protein that results is implicated in various malignancies, including lung adenocarcinoma, mucinous adenoma, ductal carcinoma of the pancreas and colorectal carcinoma. Alternative splicing leads to variants encoding two isoforms that differ in the C-terminal region. [provided by RefSeq, Jul 2008] |
| Primary Cell Localization | Cytoplasm |
| ObjectType | Protein |

---

|  |  |
| --- | --- |
| Pathway | Genes with Mutations Associated with Aging in Humans |
|  | Osteoarthritis |
|  | Oncogene Induced Cellular Senescence |
|  | mTOR Signaling |
|  | mTOR Signaling Hyperfunction |
|  | SIRT2 Signaling in Aging |
|  | Insulin Signaling |
|  | Toll-like Receptor Independent Sterile Inflammation |
|  | CHRNA7 -> NOS1 Production |
|  | RAGE/AGER and S100 Proteins in Cardiovascular Injury |
|  | Arterial Hypertension |
|  | Endothelial Cell Dysfunction in Arterial Hypertension |
|  | Smooth Muscle Cell Dysfunction in Arterial Hypertension |
|  | Myocardial Remodeling |
|  | Myocardial Infarction |
|  | Proteins Involved in Melanoma |
|  | Dendritic Cells Function in Psoriasis |
|  | Advanced Glycation End Products Pathway in Diabetic Microangiopathy |
|  | Peripheral Tissue Microangiopathy |
|  | Tubular Cell Dysfunction in Progressive Diabetic Nephropathy |
|  | Podocyte Dysfunction in Diabetic Nephropathy |
|  | Endothelial Cell Dysfunction in Early Diabetic Nephropathy |
|  | Mesangial Cell Dysfunction in Diabetic Nephropathy |
|  | Microangiopathy in Diabetic Neuropathy |
|  | Proliferative Diabetic Retinopathy |
|  | AGE Related Insulin Resistance in Skeletal Muscles |
|  | Proteins Involved in Colorectal Neoplasms |
|  | Cetuximab Resistance in Colorectal Cancer |
|  | CagA Phosphorylation Dependent Signaling |
|  | FLT3/KIT/MLL Signaling in Leukemia |
|  | Proteins Involved in Acute Myeloid Leukemia |
|  | Apoptosis Block in Acute Myeloid Leukemia Cell |
|  | Acute Myeloid Leukemia |
|  | Myeloid Cell Differentiation Block in Leukemia |
|  | Multiple Myeloma |
|  | Proteins Involved in T-Cell Acute Lymphoblastic Leukemia |
|  | Proteins Involved in Hepatocellular Carcinoma |
|  | Hepatocellular Carcinoma |
|  | Growth Factor Signaling in Hepatocellular Carcinoma |
|  | Proteins Involved in Pancreatic Cancer |
|  | Pancreatic Ductal Carcinoma |
|  | Growth Factor Signaling in Pancreatic Neoplasms |
|  | Pancreatic Neuroendocrine Tumors |
|  | TGFBR Signaling in Pancreatic Neoplasms |
|  | Dendritic Cell Dysfunction in Psoriatic Arthritis |
|  | Diabetes Induced Periodontitis |
|  | Mevalonate Kinase Deficiency |
|  | Proteins Involved in Glioblastoma |
|  | Proteins Involved in Glioma |
|  | SPP1 Induced Survival of Myelin Specific T-Cells in Multiple Sclerosis |
|  | Endometrial Cancer |
|  | Endometrioid Endometrial Cancer |
|  | Proteins Involved in Endometrial Cancer |
|  | Clear Cell Endometrial Cancer and Papillary Serous Endometrial Cancer |
|  | Genes with Mutations Associated with Endometrial Cancer |
|  | Proteins Involved in Endometriosis |
|  | Endometrioid Ovarian Carcinoma |
|  | Proteins Involved in Endometrioid Ovarian Carcinoma |
|  | Genes with Mutations Associated with Ovarian Cancer |
|  | Clear Cell Ovarian Carcinoma |
|  | Proteins Involved in Mucinous Ovarian Carcinoma |
|  | EGFR and PTAFR Signaling in Ovarian Cancer |
|  | High-Grade Serous Ovarian Carcinoma |
|  | Low-Grade Serous Ovarian Carcinoma |
|  | Proteins Involved in Low-Grade Serous Ovarian Carcinoma |
|  | Ovarian Cancer |
|  | Mucinous Ovarian Carcinoma |
|  | HMGB1/RAGE Signaling in Middle Ear Cholesteatoma |
|  | Cholesteatoma |
|  | Immune System Role in Otitis Media |
|  | Alveolar Macrophages Dysfunction in COPD |
|  | T-Cell Cytotoxic Activation |
|  | Alveolar Epithelial Cell Dysfunction in COPD |
|  | Advanced Glycation End Products in Diabetes or Age Related Erectile Dysfunction |
|  | S100P Expression Target |
|  | HMGB1 Expression Targets |
|  | TTR Expression Targets |
|  | S100A Expression Targets |
|  | S100B Expression Targets |
|  | Acetylcholine Expression Targets |
|  | CHRNA7 -> CREB Signaling |
|  | Proteins with Altered Expression in Cancer-Associated Sustaining of Proliferative Signaling |
|  | Genes with Mutations in Cancer-Associated Sustaining of Proliferative Signaling |
|  | NFKB Canonical Signaling Activation in Cancer |
|  | RAS/RAF/MAPK Signaling Activation in Cancer |
|  | RAS/RAF/MAPK Signaling Activation by Blocking of Tumor Suppressors |
|  | VEGFA Dependent Angiogenesis in Cancer |
|  | Metabolic Effects of Oncogenes and Tumor Suppressor in Cancer Cells |
|  | Genes with Mutations in Cancer Metabolic Reprogramming |
|  | Proteins with Altered Expression in Cancer Metabolic Reprogramming |
|  | IDO1 in Cancer Immune Escape |
|  | AGER -> NF-kB Signaling |
|  | AGER -> CREB/SP1 Signaling |
|  | FOXO1 Signaling |
|  | KRAS Signaling |
|  | Estrogens/ESR1 non-Genomic Signaling |
|  | Model of PPARG signaling in psoriasis |
|  | Model of PPARG related pathways in psoriasis (short version) |
|  | H2A-K120, H2A-K119 and H3-K4 |
|  | Epigenetic Modifications of H2A-K120, H2A-K119 and H3-K4 |
|  | 1\_Local estrogen production stimulates ectopic endometrium proliferation |
|  | 3\_Arterial wall inflammation and calcification in atherosclerosis |
|  | 1\_Cigarette smoke causes dysfunction and production of inflammatory mediators in alveolar epithelial cells |
|  | 3\_Role of alveolar macrophages in pulmonary tissue destruction in COPD |
|  | 4\_1\_Polymorphisms associated with inflammatory bowel diseases |
|  | 1\_Microangiopathy in diabetic neuropathy |
|  | 2\_Neuron and Schwann cell death |
|  | drug-target molsynthesis |
|  | Model of PPARG signaling in psoriais (tested) |
|  | public list |
|  | New Pathway |
|  | KRAS->RAFs->MAPK1/3 |

---

|  |  |
| --- | --- |
| Group | Genes with Mutations Associated with Colorectal Cancer |
|  | Genes Associated with Ulcerative Colitis |
|  | Genes with Mutations Associated with Acute Myeloid Leukemia |
|  | Genes with Mutations Associated with Multiple Myeloma |
|  | Genes with Mutations Associated with Hepatocellular Carcinoma |
|  | Genes with Mutations Associated with Pancreatic Cancer |
|  | Proteins Involved in Cowden Syndrome |
|  | Genes Associated with Syndromic Epilepsy |
|  | Genes with Mutations Associated with Breast Cancer |
|  | Genes with Mutations Associated with Endometrial Cancer |
|  | Genes with Mutations Associated with Ovarian Cancer |

---

|  |  |
| --- | --- |
| MedScan ID | 3845 |

---

|  |  |
| --- | --- |
| LocusLink ID | 3845 |
|  | 16653 |
|  | 24525 |

---

|  |  |
| --- | --- |
| Alias | Kras2 |
|  | c-K-ras2 |
|  | AI929937 |
|  | K-RAS2A |
|  | Kirsten rat sarcoma oncogene 2, expressed |
|  | c-K-ras |
|  | K-RAS4B |
|  | OTTMUSP00000025007 |
|  | GTPase KRas |
|  | v-Ki-ras2 Kirsten rat sarcoma 2 viral oncogene homolog |
|  | K-Ras 2B |
|  | K-ras |
|  | c-Kirsten-ras protein |
|  | KRAS |
|  | KRAS proto-oncogene, GTPase |
|  | v-Ki-ras2 Kirsten rat sarcoma viral oncogene homolog |
|  | kras1 |
|  | Kirsten rat sarcoma viral oncogene homolog |
|  | Kirsten rat sarcoma viral oncogene homologue 2 (active) |
|  | MGC7141 |
|  | c-Ki-ras2 |
|  | c-K-ras2 protein isoform b |
|  | c-K-ras proteins |
|  | K-ras p21 |
|  | K-RasB |
|  | p21b |
|  | Kirsten rat sarcoma viral oncogene |
|  | OTTMUSP00000025006 |
|  | oncogene KRAS2 |
|  | transforming protein p21 |
|  | Kirsten rat sarcoma-2 viral (v-Ki-ras2) oncogene homolog |
|  | K-RASA |
|  | cellular c-Ki-ras2 proto-oncogene |
|  | RP24-359O2.1 |
|  | Kirsten rat sarcoma viral proto-oncogene |
|  | Ki-Ras |
|  | K-Ras 2 |
|  | K-RAS4A |
|  | PR310 c-K-ras oncogene |
|  | c-Kirsten-ras proto-oncogene |
|  | RASK2 |
|  | c-K-ras2 protein isoform a |
|  | c-Ki-ras |
|  | Transforming protein p21b |
|  | NS |
|  | NS3 |
|  | CFC2 |
|  | RALD |
|  | K-RAS2B |
|  | K-ras p21 protein |
|  | cellular transforming proto-oncogene |
|  | ras |
|  | Kras-2 |
|  | p21 protein |
|  | p21 |
|  | c-K-ras2 protein |

---

|  |  |
| --- | --- |
| GO ID | 0019003 |
|  | 0019002 |
|  | 0005525 |
|  | 0003924 |
|  | 0030275 |
|  | 0044877 |
|  | 0000165 |
|  | 0007265 |
|  | 0030036 |
|  | 0019221 |
|  | 0038002 |
|  | 0060441 |
|  | 0007565 |
|  | 0021897 |
|  | 0048873 |
|  | 0001889 |
|  | 0045596 |
|  | 0043524 |
|  | 0043406 |
|  | 0051092 |
|  | 0035022 |
|  | 0008284 |
|  | 2000774 |
|  | 0010628 |
|  | 0051000 |
|  | 0001934 |
|  | 0048169 |
|  | 0031647 |
|  | 0032228 |
|  | 0051384 |
|  | 0035900 |
|  | 0051385 |
|  | 0002223 |
|  | 0051146 |
|  | 0008542 |
|  | 0005737 |
|  | 0005829 |
|  | 0031234 |
|  | 0005925 |
|  | 0016020 |
|  | 0045121 |
|  | 0005739 |
|  | 0005886 |
|  | 0000166 |
|  | 0007165 |
|  | 0005622 |
|  | 0032403 |
|  | 0006913 |
|  | 0045087 |
|  | 0006886 |
|  | 0000186 |
|  | 0005515 |
|  | 0007173 |
|  | 0038095 |
|  | 0007411 |
|  | 0008543 |
|  | 0048010 |
|  | 0048011 |
|  | 0038128 |
|  | 0035176 |
|  | 0007264 |
|  | 0050900 |
|  | 0006184 |
|  | 0008286 |
|  | 0007596 |
|  | 0005624 |
|  | 0007569 |
|  | 0006897 |

---

|  |  |
| --- | --- |
| KEGG ID | hsa:3845 |
|  | mmu:16653 |
|  | rno:24525 |

---

|  |  |
| --- | --- |
| Organism | Homo sapiens {Organism urn:agi-taxid:9606} |
|  | Mus musculus {Organism urn:agi-taxid:10090} |
|  | Rattus norvegicus {Organism urn:agi-taxid:10116} |
|  | Homo sapiens |
|  | Mus musculus |
|  | Rattus norvegicus |

---

|  |  |
| --- | --- |
| Mouse chromosome position | 6 77.37 cM |
|  | 6 71.2 cM |

---

|  |  |
| --- | --- |
| OMIM ID | 190070 |
|  | 601626 |
|  | 109800 |
|  | 260350 |
|  | 615278 |
|  | 108010 |
|  | 163200 |
|  | 114480 |
|  | 137215 |
|  | 211980 |
|  | 609942 |
|  | 614470 |
|  | 115150 |
|  | 218040 |

---

|  |  |
| --- | --- |
| Rat chromosome position | 4q44 |

---

|  |  |
| --- | --- |
| Hugo ID | 6407 |
|  | HGNC:6407 |

---

|  |  |
| --- | --- |
| Human chromosome position | 12p12.1 |

---

|  |  |
| --- | --- |
| Swiss-Prot Accession | A0A024RAV5 |
|  | P01116 |
|  | I1SRC5 |
|  | L7RSL8 |
|  | P01116.1 |
|  | P32883 |
|  | Q5J7N1 |
|  | Q0VDV7 |
|  | P32883.1 |
|  | A0JN17 |
|  | P08644 |
|  | P08644.3 |
|  | A8K8Z5 |
|  | B0LPF9 |
|  | P01118 |
|  | Q96D10 |
|  | P04200 |
|  | P08643 |
|  | P46203 |
|  | P97914 |
|  | Q14014 |
|  | Q14015 |
|  | Q15285 |
|  | Q71SP6 |
|  | Q7KZ06 |
|  | Q92668 |
|  | Q3U2W7 |
|  | Q53WZ3 |

---

|  |  |
| --- | --- |
| PIR ID | A93311 |
|  | B93311 |
|  | A01365 |
|  | B01365 |
|  | I58402 |

---

|  |  |
| --- | --- |
| GenBank ID | NC\_000012 |
|  | NM\_001369787 |
|  | NP\_001356716 |
|  | NM\_001369786 |
|  | NP\_001356715 |
|  | NM\_004985 |
|  | NP\_004976 |
|  | NM\_033360 |
|  | NP\_203524 |
|  | NG\_007524 |
|  | AC087239 |
|  | AC092794 |
|  | AF285779 |
|  | AAF91482 |
|  | AH001529 |
|  | AAA36554 |
|  | AH005283 |
|  | AAB59444 |
|  | AAB59445 |
|  | CH471094 |
|  | EAW96511 |
|  | EAW96512 |
|  | EAW96513 |
|  | EAW96514 |
|  | EF471933 |
|  | ABP96992 |
|  | EF471950 |
|  | ABP97009 |
|  | EF471954 |
|  | ABP97013 |
|  | EF471956 |
|  | ABP97015 |
|  | EF685661 |
|  | ABS10683 |
|  | EF685662 |
|  | ABS10684 |
|  | EU332849 |
|  | ABY87538 |
|  | HC464412 |
|  | CBK51852 |
|  | HC464568 |
|  | CBK51925 |
|  | HD032929 |
|  | CBV35640 |
|  | HM459605 |
|  | ADQ00188 |
|  | JC006857 |
|  | CDL93479 |
|  | JQ248012 |
|  | AFF18187 |
|  | JX512447 |
|  | AGC09594 |
|  | KF459678 |
|  | KX302153 |
|  | KY752515 |
|  | ASO66780 |
|  | KY828926 |
|  | ASO66781 |
|  | LC090171 |
|  | LC090172 |
|  | LC090174 |
|  | LC090175 |
|  | LC090177 |
|  | LC090179 |
|  | LC090180 |
|  | LC090181 |
|  | LC090182 |
|  | LC090184 |
|  | LC090187 |
|  | LC090191 |
|  | LC090192 |
|  | LC090193 |
|  | LC090194 |
|  | M26261 |
|  | AAA36148 |
|  | M34904 |
|  | AAA36149 |
|  | MF083614 |
|  | ASO66782 |
|  | S64261 |
|  | AAD13953 |
|  | X01669 |
|  | CAA25828 |
|  | X02825 |
|  | CAA26593 |
|  | AA812162 |
|  | AF493917 |
|  | AAM12631 |
|  | AI539465 |
|  | AK292510 |
|  | BAF85199 |
|  | AW117295 |
|  | BC010502 |
|  | BC013572 |
|  | AAH13572 |
|  | BC029545 |
|  | BG258021 |
|  | BT007153 |
|  | AAP35817 |
|  | CD245555 |
|  | M17087 |
|  | AAA36555 |
|  | M35504 |
|  | AAA35689 |
|  | M35505 |
|  | AAA35690 |
|  | M54968 |
|  | AAB41942 |
|  | P01116 |
|  | NC\_000072 |
|  | XM\_006506919 |
|  | XP\_006506982 |
|  | XM\_006506918 |
|  | XP\_006506981 |
|  | NM\_021284 |
|  | NP\_067259 |
|  | NT\_166305 |
|  | AC157091 |
|  | CH466572 |
|  | EDL10686 |
|  | CU207316 |
|  | CU458742 |
|  | JC006855 |
|  | CDL93478 |
|  | K01927 |
|  | AAA40037 |
|  | M16708 |
|  | S39586 |
|  | AAO46052 |
|  | X02452 |
|  | CAA26295 |
|  | X02453 |
|  | X02454 |
|  | X02455 |
|  | CAA26296 |
|  | X02456 |
|  | X15887 |
|  | CAA33897 |
|  | AK155064 |
|  | BAE33023 |
|  | AK164065 |
|  | BAE37609 |
|  | AY487807 |
|  | AAS58056 |
|  | BC004642 |
|  | AAH04642 |
|  | BC010202 |
|  | AAH10202 |
|  | BC014700 |
|  | BC031623 |
|  | BC119495 |
|  | AAI19496 |
|  | BC141051 |
|  | AAI41052 |
|  | U76425 |
|  | U76426 |
|  | X00485 |
|  | CAA25160 |
|  | P32883 |
|  | NC\_005103 |
|  | XM\_008763354 |
|  | XP\_008761576 |
|  | XM\_008763355 |
|  | XP\_008761577 |
|  | NM\_031515 |
|  | NP\_113703 |
|  | XM\_017592442 |
|  | XP\_017447931 |
|  | XM\_017592443 |
|  | XP\_017447932 |
|  | AC\_000072 |
|  | AABR07062484 |
|  | AAHX01033713 |
|  | AAHX01033714 |
|  | AH002242 |
|  | AAA42011 |
|  | CH473964 |
|  | EDM01439 |
|  | M54870 |
|  | AAA40937 |
|  | BC126086 |
|  | AAI26087 |
|  | FQ228206 |
|  | FQ230946 |
|  | FQ232665 |
|  | U09793 |
|  | AAB60458 |
|  | P08644 |
|  | XP\_006536102 |
|  | XP\_006536103 |
|  | X01228 |
|  | M12260 |
|  | XP\_006237717 |
|  | AC\_000028 |
|  | XP\_006719132 |
|  | XM\_006237655 |
|  | NC\_018923 |
|  | XM\_008763353 |
|  | XM\_008763352 |
|  | AABR06034275 |
|  | XP\_008761575 |
|  | XP\_008761574 |
|  | ABBA01068017 |
|  | ABBA01068018 |
|  | AMYH02026221 |
|  | AMYH02026220 |
|  | DQ890649 |
|  | AAHY01058889 |
|  | K03210 |
|  | XM\_006536039 |
|  | CAQ52125 |
|  | CAQ52126 |
|  | CAQ52124 |
|  | XM\_006536040 |
|  | ABM81575 |
|  | XM\_006719069 |
|  | L00049 |
|  | L00048 |
|  | L00047 |
|  | L00046 |
|  | L00045 |
|  | L00044 |
|  | AC\_000144 |
|  | XM\_011520653 |
|  | XM\_005253365 |
|  | XP\_011518955 |
|  | XP\_005253422 |
|  | DQ893829 |
|  | ABM84755 |
|  | K03209 |
|  | CAQ52105 |
|  | CAQ52106 |
|  | NT\_009714 |
|  | NW\_925328 |
|  | NW\_001838052 |
|  | AC\_000055 |
|  | CR625034 |
|  | Q14014 |
|  | Q14015 |
|  | Q15285 |
|  | Q71SP6 |
|  | Q7KZ06 |
|  | Q92668 |
|  | NT\_039360 |
|  | NW\_001030820 |
|  | Q0VDV7 |
|  | Q3U2W7 |
|  | Q53WZ3 |
|  | Q5J7N1 |
|  | NW\_047696 |
|  | NW\_001084832 |

---

|  |  |
| --- | --- |
| Swiss-Prot ID | RASK\_HUMAN |
|  | RASK\_MOUSE |
|  | RASK\_RAT |
|  | Q5J7N1\_MOUSE |
|  | A0JN17\_RAT |
|  | A8K8Z5\_HUMAN |
|  | B0LPF9\_HUMAN |
|  | Q3U2W7\_MOUSE |

---

|  |  |
| --- | --- |
| Cell Localization | Cell membrane |
|  | Cytoplasm |
|  | Cytosol |
|  | Cytoplasm, cytosol |

---

|  |  |
| --- | --- |
| Ensembl ID | ENSG00000133703 |
|  | ENSP00000308495.3 |
|  | ENST00000311936.7 |
|  | ENSP00000256078.4 |
|  | ENST00000256078.8 |
|  | ENSMUSG00000030265 |
|  | ENSMUSP00000032399.5 |
|  | ENSMUST00000032399.11 |
|  | ENSMUSP00000107339.1 |
|  | ENSMUST00000111710.7 |
|  | ENSRNOG00000009338 |
|  | ENSRNOP00000012887.2 |
|  | ENSRNOT00000012887.6 |
|  | ENSRNOP00000012588.2 |
|  | ENSRNOT00000012588.4 |
|  | ENSP00000308495 |
|  | ENSMUSP00000032399 |
|  | ENSRNOP00000012887 |
|  | ENSRNOP00000012588 |
|  | ENSMUST00000032399 |
|  | ENSMUSP00000107339 |
|  | ENST00000311936 |
|  | ENSP00000256078 |
|  | ENSRNOT00000012588 |
|  | ENSMUST00000111710 |
|  | ENSRNOT00000012887 |
|  | ENST00000256078 |

---

|  |  |
| --- | --- |
| MGI ID | MGI:96680 |
|  | 96680 |

---

|  |  |
| --- | --- |
| RGD ID | 2981 |

---

|  |  |
| --- | --- |
| Unigene ID | Hs.505033 |
|  | Mm.383182 |
|  | Rn.24554 |

---

|  |  |
| --- | --- |
| Homologene ID | 37990 |

---

|  |  |
| --- | --- |
| IPI ID | IPI00134941 |
|  | IPI00423568 |
|  | IPI00210097 |
|  | IPI00203826 |
|  | IPI00113248 |
|  | IPI00423570 |
|  | IPI00790968 |
|  | IPI00791238 |
|  | IPI00845559 |
|  | IPI00830951 |
|  | IPI00857121 |

---
